# COVID-19 and New-Onset Psychosis: A Comprehensive Review

**DOI:** 10.3390/jpm13010104

**Published:** 2023-01-02

**Authors:** Lorenzo Moccia, Georgios D. Kotzalidis, Giovanni Bartolucci, Sara Ruggiero, Laura Monti, Marco Biscosi, Beatrice Terenzi, Ottavia M. Ferrara, Marianna Mazza, Marco Di Nicola, Delfina Janiri, Alessio Simonetti, Emanuele Caroppo, Luigi Janiri, Gabriele Sani

**Affiliations:** 1 Department of Psychiatry, Fondazione Policlinico Universitario Agostino Gemelli IRCCS, L.go Agostino Gemelli 8, 00168 Rome, Italy; 2 Department of Neuroscience, Section of Psychiatry, Università Cattolica del Sacro Cuore, 00168 Rome, Italy; 3 Department of Neurosciences, Mental Health, and Sensory Organs (NESMOS), Sapienza University of Rome, Sant’Andrea Hospital, Via di Grottarossa 1035-1039, 00189 Rome, Italy; 4 Department of Mental Health, Local Health Authority ROMA 2, 00159 Rome, Italy

**Keywords:** schizophrenia, bipolar disorder, inflammation, delirium, side effects, viral infection

## Abstract

Psychosis is a multifactorial condition that typically involves delusions, hallucinations, and disorganized thought, speech or behavior. The observation of an association between infectious epidemics and acute psychosis dates back to the last century. Recently, concerns have been expressed regarding COVID-19 and the risk for the development of new-onset psychosis. This article reviewed the current evidence of a possible link between SARS-CoV-2 and risk of psychosis as an acute or post-infectious manifestation of COVID-19. We here discuss potential neurobiological and environmental factors as well as a number of challenges in ascribing a causal pathogenic relationship between SARS-CoV-2 infection and new-onset psychosis.

## 1. Introduction

Psychosis is a highly disruptive condition that typically involves delusions, hallucinations, and disorganized thought, speech or behavior [1]. As with many mental disorders, psychosis can be triggered by several different causes, including psychiatric, neurodevelopmental, neurologic, and medical conditions [2,3]. The COVID-19 pandemic caused by the novel coronavirus SARS-CoV-2 had a profound impact on mental health [4]. A large number of individuals, whether affected by the infection or unaffected, developed anxiety and depression symptoms, but the former at a higher percentage [5]. Psychiatric and neurological sequelae were largely reported after acute infection. Recent evidence highlights that a relevant percentage of patients infected with SARS-CoV-2 may display symptoms of anxiety and depression [6], of obsessive–compulsive disorder (OCD) [7], post-traumatic stress disorder (PTSD) [8], sleep disturbances, cognitive impairment, fatigue [9], mood changes, and delirium [10]. Though infrequently, cases of new-onset psychosis have been observed in patients with COVID-19 since the beginning of the pandemic [11], leading to concerns that SARS-CoV-2 infection may be associated with an increased risk for the development of psychosis. However, the validity of this association has been questioned [12].

## 2. Viral Infections and Risk for Psychosis

The documented connection between viral epidemics and psychosis can be traced back to more than a century ago, when several cases of acute post-influenzal psychosis were reported during the 1918 Spanish flu pandemic. In a pioneering writing published in 1919, Karl Menninger described a cohort of patients presenting symptoms of “dementia praecox” and other disturbances, including delusions, confusion, and psychomotor agitation; patients were admitted to the Boston Psychopathic Hospital from 15 September 1918, through 15 December 1918, at the peak of Spanish flu pandemic in New England [13]. Menninger made a considerable effort to distinguish these patients from those who presented with psychotic symptoms as part of a more generalized impairment of consciousness or delirium. In 1926, Menninger published a follow-up study of 50 patients diagnosed with dementia praecox after the 1918 influenza outbreak [14]. He was astonished to recognize that the majority of the cases previously diagnosed as dementia praecox had completely recovered at follow-up, an observation that was in contrast with the Kraepelinian notion that dementia praecox patients, later termed schizophrenia (SCZ), were expected to experience a chronic and deteriorating clinical course.

During other viral flu-like pandemics, including the 2003 SARS-CoV-1, the 2009 H1N1, and the 2012 MERS outbreaks, data suggesting an increased incidence of psychosis were obtained [15]. A review of the literature, conducted by Brown et al. [16], estimated that around 0.9–4% of individuals exposed to H1N1 influenza, Ebola, SARS, MERS, or COVID-19 developed psychosis or psychotic symptoms, a significantly higher percentage than the median incidence of 0.015% observed among the general population. Similar observations have been made in the context of the exposure to several other viral agents, including herpes simplex virus, human immunodeficiency virus (HIV), Epstein–Barr virus, and cytomegalovirus [17,18].

## 3. COVID-19 and New-Onset Psychosis

Despite the fact that the incidence of psychosis appears to increase in the general population following historical viral pandemics, to date there is still relatively limited evidence linking psychosis and SARS-CoV-2. Reports of new-onset psychosis in individuals presenting with either current COVID-19 or previous SARS-CoV-2 infection have been described, but have been beset with several issues, including inadequate sample size and lack of attention to potential confounding factors.

A retrospective cohort study investigating neurological and psychiatric sequelae among 236,379 patients with COVID-19 over a 6-month follow-up reported an estimated incidence of 0.42% for a first diagnosis of psychotic disorder as well as a significantly increased hazard risk (HR) of presenting psychotic symptoms compared to patients with influenza (HR 2.27) or other respiratory tract infections (HR 1.49) in the same period [19]. However, caution should be made when interpreting these results in the light of possible confounders, especially among patient with COVID-19 who required hospitalization, including misdiagnosis of delirium [11,20], undocumented previous mental illness, and iatrogenic factors [16]. To tackle this issue, Chaudhary et al. [21] conducted a systematic review targeting case reports and case series that evaluated the occurrence of new-onset psychosis or exacerbation of clinically stable psychosis among patients with COVID-19. Studies involving patients with delirium, encephalitis, or steroid use, as well as other medical conditions that could have contributed to psychotic symptoms, were excluded. Fifty-seven unique cases were included of whom 66.7% had no previous psychiatric history. Most patients were above the typical age of onset for psychosis with a mean age at onset of 43.4 years for men and 40.3 years for women. The majority of patients had mild COVID-19-related respiratory illnesses, neurological, or psychotic symptoms, with around 26.3% of patients presenting with moderate-to-severe COVID-19-related disease and complications. Delusions and hallucinations were the most common psychotic symptoms. Most patients responded to low-to-moderate antipsychotic doses with a complete recovery observed in about 72% of the sample.

## 4. Potential Etiological Pathways Linking SARS-CoV-2 Infection and New-Onset Psychosis

The Coronavirus Disease 2019 (COVID-19) represents a severe multiorgan pathology that, in addition to cardio-respiratory manifestations, may affect the function of the central nervous system (CNS) [22]. SARS-CoV-2, similarly to other respiratory viruses, may damage the CNS via both direct invasion and immune activation. SARS-CoV-2 infects the epithelial cells of lungs and gastrointestinal tract as primary targets. It gains access into cells following binding to plasmalemmal ACE2 enzyme with subsequent endocytic internalization [23]. The endocytosis of the ACE2–virus complex leads to a depletion of the plasmalemmal pool of ACE2 with a secondary reduction in conversion of angiotensin II to angiotensin 1–7; the latter peptide possesses marked anti-inflammatory properties, and its reduction significantly contributes to lung failure and to the massive occurrence of pulmonary fibrosis observed among patients with COVID-19. The ACE2 enzyme is also significantly expressed in other tissues, including heart, kidney, endothelium, and the CNS [24,25]. As a consequence, SARS-CoV-2 can reach multiple organs. The virus can enter the brain through several routes. It can migrate through the axons of many nerves, including the olfactory, trigeminal, and vagus nerves. SARS-CoV-2 can also access the CNS by infecting endothelial cells of cerebral vessels or by invading perivascular spaces of the glymphatic system [26,27]. At the CNS level, the ACE2 expressing neural cells include dopaminergic and serotonergic nuclei, glutamatergic neurons, and the lateral ventricles. Furthermore, in the substantia nigra, the percentage expression of ACE2 is comparable to that of the lungs [28]. All these brain regions are neurochemically and structurally involved in SCZ and while damage to these CNS sites may not necessarily reflect causation; their relative vulnerability to SARS-CoV-2 infection may play a role in shaping future psychopathology [29]. Localization matters in this respect, inasmuch penetration of the virus in circumventricular organs may confer vulnerability to many neuropsychiatric outcomes [30]. In addition, ACE2 exhibits significant genetic co-expression with dopamine decarboxylase, an enzyme involved in dopamine and serotonin synthetic pathways [31]. Accordingly, it has been suggested that SARS-CoV-2-induced downregulation of ACE2 expression may be paralleled by alterations of both dopamine and serotonin synthesis [32], possibly leading to psychiatric sequelae.

COVID-19-related CNS dysfunction may also result from systemic inflammation, often known as systemic inflammatory response syndrome or “cytokine storm”. In the context of COVID-19, the massive increase in circulating pro-inflammatory factors may deeply affect the blood–brain barrier (BBB) integrity, allowing inflammatory cells and molecules to access the CNS. Neuroinflammation perturbs brain homeostasis, alters neural networks, and eventually induces neuronal deaths [33]. Both the infection itself and hypoxia stimulate cytokine release, which can increase BBB permeability. Cerebral hypoxia may activate key inflammatory transcription factors, resulting in an overproduction of pro-inflammatory messengers as well as in an excessive glial reactivity which further contributes to the loss of synapses and neurons [34]. In addition, circulating pro-inflammatory factors may enhance hypothalamic–pituitary–adrenal (HPA) axis activity, which contributes to sustaining and promoting neuroinflammation due to glucocorticoid increase [35]. Furthermore, though SARS-CoV-2 is rarely found in the cerebrospinal fluid [36], viral-induced immune reactions and autoimmunity (during or after the acute infection) may provide another route by which SARS-CoV-2 can impact CNS function [33].

Systemic inflammation has long been recognized as a potential immune-related trigger in the pathogenesis of neuropsychiatric manifestations associated with viral infections [37]. According to a vulnerability–stress–inflammation hypothesis of psychotic illness, stress during both pre- and postnatal neurodevelopment may prime the individual for an abnormal response to future stressors, typically during adolescence or early adulthood [29]. Biological stressors, such as viral infections and immune activation, may affect the HPA axis, whose dysregulation contributes to some abnormalities observed in SCZ, including increased baseline cortisol and reduced hippocampal volume, as well as disturbances in dopamine and glutamate transmission [38,39]. In addition, microglia may release pro-inflammatory factors in response to stress or infection and, similarly to the HPA axis, can be primed by neurodevelopmental stressors [40]. Notably, overactivation of microglial cells may be involved in the pathophysiology of SCZ, leading to abnormal synaptic pruning and to altered neurotransmitter metabolism secondary to augmented cytokine release [41]. Patients with COVID-19 and associated psychotic symptoms may show an increased level of pro-inflammatory cytokines, including IL-6, TNF-α, IL-1β, CRP, ferritin, LDH, and D-Dimer [42,43,44,45]. Likewise, an upregulation of pro-inflammatory factors seems to characterize drug-naïve patients in their first episode of psychosis [46]. Furthermore, there is evidence for a longitudinal association between increased C-reactive protein (CRP) serum levels in adolescence and diagnosis of psychotic spectrum disorders in adulthood [47].

Autoimmunity may also be implicated in the pathogenesis of psychotic illness [48]. Accordingly, there is evidence for both shared genetic risk factors between several immune diseases and SCZ [49], and for an increased risk of developing psychotic spectrum disorders in individuals with autoimmune illnesses and severe infections that required admission to hospital [50]. In the context of SARS-CoV-2 infection, several cases of patients with COVID-19 and associated CNS autoimmune demyelinating disease have been reported [51]. Notably, several demyelinating disorders are related to the presence of psychotic symptoms and, according to evidence, reduced CNS white matter integrity secondary to demyelination may play a role in the pathophysiology of SCZ [52].

## 5. COVID-19 and New-Onset Psychosis: Possible Confounders

Causality is a condition required to determine the association between COVID-19 and new-onset psychosis [12]. This issue may prove to be challenging for both clinicians and researchers. Considering the summary of available evidence, the assumption of several studies targeting the association between COVID-19 and new-onset psychosis does not meet the Bradford Hill criteria of strength, consistency, specificity, or temporality, required for a confident determination of causality [53]. However, in the light of existing knowledge, there is biological plausibility supporting this association. Several confounders need to be addressed when examining any case for neuropsychiatric sequelæ in patients with COVID-19, including psychosocial and iatrogenic factors [12]. The association between psychosis and a range of psychosocial factors, including stressful life events, has been extensively explored, suggesting that psychosocial stressors are an important risk factor for both the onset and the exacerbation of symptoms [54,55]. On the one hand, individuals with COVID-19 suffered various stressors during the pandemic, including stress related to the quarantine, issues related to the treatment environment, and limited information about COVID-19 [56,57]. On the other hand, social stress can affect brain function, and in particular the molecular targets involved in psychosis, including dopaminergic signaling [58].

As regards iatrogenic factors, the neuropsychiatric side effects of treatments should be considered. Several drugs have been adopted empirically, especially in the first phase of the pandemic, in the treatment of COVID-19, including antibiotics, antivirals, antimalarials, and corticosteroids [59]. Even though antibiotics are not indicated as a treatment for SARS-CoV-2 infection, the four most frequently prescribed therapeutic classes in the first wave of the pandemic were azithromycin (50.7%), doxycycline (13.0%), amoxicillin (9.4%), and levofloxacin (6.7%) [60]. Notably, there is evidence suggesting a direct relationship between acute psychosis and antibiotic exposure, with macrolides and fluoroquinolones presenting the greatest increased odds of psychosis [61]. Similarly, for the antimalarial hydroxychloroquine, which was one of the most promising therapies tested in patients with COVID-19 at the beginning of the pandemic, a documented association with neuropsychiatric adverse effect, including acute psychosis, is well established [62,63,64,65]. Hydroxychloroquine typically crosses the blood–brain barrier to concentrate within the CNS [66,67,68,69]. Several mechanisms have been proposed as contributing to the neuropsychiatric side effects of chloroquine, including increased dopaminergic activity, NMDA excitotoxicity, GABAergic inhibition, and lysosomal dysfunction [70]. Transient, acute psychosis is a well-documented, long-known [71,72] adverse effect of corticosteroids that usually occurs with systemic steroid treatment [73,74,75,76], but it may also occur with topical application [77] and be long-lasting [78]. Findings coming from the previous SARS outbreak highlighted that patients on higher doses of corticosteroids had an increased risk of psychosis [16].

## 6. Case Presentation

Based on this evidence, we present here the cases of two patients admitted to the outpatient psychiatric service of the Fondazione Policlinico Universitario Agostino Gemelli IRCCS (Rome, Italy) between 1 July 2020 and 1 July 2021 with new-onset psychosis and delusions, and who had reported a history of recent SARS-CoV-2 infection.

### 6.1. Case 1

A 48-year-old woman was diagnosed with bilateral pneumonia and mild respiratory insufficiency related to SARS-CoV-2 infection. She had been admitted to the hospital in mid-April 2020. Blood analyses showed an inflammatory state, including elevated serum C-reactive protein and leukocytosis. The patient was treated with high-flow oxygen as well as with oral lopinavir–ritonavir, hydroxychloroquine, and ibuprofen. After three weeks of hospitalization, once the patient’s medical conditions had improved considerably and the SARS-CoV-2 nasal/oropharyngeal swab was negative, she was discharged. In June 2020, she presented to our emergency department (ED) accompanied by two family members, complaining somatic delusions concerning widespread pains and internal organ failure, initial insomnia, and psychomotor retardation. She tested negative on nasal/oropharyngeal swab. At the time the patient came to ED, she presented with no psychiatric or family history and did not take any medication. Laboratory tests were normal, and no psychotropic substances were present in her urine drug screen. She was admitted to an internal medicine ward for further investigation. After admission, the patient became mute, unresponsive to external stimuli, and showed postural rigidity. Clinical electroencephalography was normal. Similarly, magnetic resonance imaging revealed no morphological or structural alterations within brain tissues and the whole ventricular system. The liaison psychiatrist suspected patient’s withdrawal was a catatonic symptom and administered a lorazepam challenge of 2 mg IV with good response. Lorazepam was administered at the dose of 2 mg PO every 8 h leading to a complete recovery from catatonic symptoms within seven days. At the subsequent psychiatric interview, the patient appeared alert and oriented as to place and time. Her mood was neutral. She reported a history of restlessness, low appetite, and olfactory hallucinations as well as delusional beliefs of being “rotten inside” shortly after her first discharge from the hospital for COVID-19. She was prescribed aripiprazole 15 mg PO daily in addition to lorazepam. Three weeks after admission, she was calm with no evidence of psychotic symptoms. She was discharged home with a diagnosis of acute and transient psychotic disorder according to the International Statistical Classification of Diseases and Related Health Problems (ICD)-10 and admitted to the outpatient psychiatric clinic for regular follow-up appointments. Lorazepam was gradually discontinued, whereas aripiprazole was reduced to 10 mg PO daily and eventually discontinued after 6 months without a return of delusional symptoms. She is currently well.

### 6.2. Case 2

A 42-year-old woman was brought to the outpatient psychiatric consulting room by her sister in July 2020. Divorced, she lived with her 18-year-old daughter, who was affected by multiple sclerosis. The patient had neither known chronic psychiatric conditions, including substance use disorders, nor was taking any long-term medications. Similarly, her family history was free from psychiatric disorders. In May 2020, she tested positive on the SARS-CoV-2 nasal/oropharyngeal swab, reporting high fever, fatigue, sore throat, and dry cough. Wishing to protect her daughter from the infection, the patient decided to move to another house in Rome. She stayed isolated for 13 days, after which she gradually recovered, though she continued suffering from anosmia and ageusia. Notably, she did not require any specific treatment for her COVID-19. At the time of the psychiatric examination, the patient reported the onset of tactile and visual hallucinations, in the form of bugs crawling on or underneath her skin, two weeks after the beginning of the infection. She washed her hands several times a day with chemical detergents and was extremely worried that the bugs may infest her daughter too, so she decided not to come back home after she recovered from COVID-19. The patient also visited a dermatologist who diagnosed a severe form of contact dermatitis and referred her to a psychiatric consultation. The patient was administered risperidone 2 mg PO daily, which was increased to 3 mg a week later. The patient showed a significant improvement in her delusional beliefs as well as an increase in her insight and judgement/reasoning abilities within one week after the onset of the treatment. Risperidone was gradually reduced to 1 mg PO during the follow-up and the patient did not show any relapse. She is about to discontinue her risperidone completely.

## 7. Concluding Remarks and Future Perspectives

The COVID-19 outbreak had a profound impact on mental health. Evidence from previous viral pandemics suggests that infectious agents, in particular respiratory viruses, may represent a risk factor for schizophrenia-like illnesses in patients who contract the infection. However, this does not necessarily mean that viral agents, including SARS-CoV-2, play a causative role in the pathogenesis of psychotic disorders. Available evidence only supports a biological plausibility for the association between COVID-19 and new-onset psychosis. The limitations of this review are inherent in the heterogeneity of methods used to assess psychosis in existing literature and in the limited number of available reports specifically addressing the issue. However, the bulk of evidence points to the possibility that viral entry in brain structures may facilitate the onset of psychotic behavior in vulnerable persons.

Clinical features of COVID-19-associated psychosis, similar to those observed in the context of several infectious diseases, include higher age at onset and response to a low-to-moderate dose of antipsychotics, as well as a quicker recovery and a generally favorable prognosis. A number of possible confounders, including iatrogenic and psychosocial factors, as well as delirium misdiagnosis, need to be addressed when examining this association. Accordingly, clinicians should pay particular attention to the possible side effects of medications used to treat COVID-19 when facing the occurrence of acute psychosis in individuals with SARS-CoV-2 infection and no prior psychiatric history. Besides this, several anti-inflammatory compounds proved to be beneficial in SCZ [79], giving rise to the opportunity of future studies targeting the use of these drugs in vulnerable cohorts affected by COVID-19. A closer look at the association between SARS-CoV-2 infection and the occurrence of psychotic symptoms may allow prevention to be better tailored and enforce intervention strategies aimed at reducing risk, taking into account the individual characteristics of each patient. This would allow a personalized approach in their treatment.

## Data Availability

Not applicable.
